# Early Adverse Perinatal Outcomes Among Women With Placental Abruption: A Multicenter Prospective Cohort Study in Uganda

**DOI:** 10.1155/jp/6074764

**Published:** 2026-09-21

**Authors:** Idiris Omar Siham, Marie Pascaline Sabine Ishimwe, Theodore Nteziyaremye, Rogers Kajabwangu, Ahmed Kiswezi Kazigo, Theoneste Hakizimana

**Affiliations:** ^1^ Department of Obstetrics and Gynecology, Kampala International University, Ishaka, Uganda, kiu.ac.ug; ^2^ Department of Pediatrics and Child Health, Kampala International University, Ishaka, Uganda, kiu.ac.ug; ^3^ Department of Sciences, University of Rwanda, Kigali, Rwanda, ur.ac.rw; ^4^ Department of Obstetrics and Gynecology, Mbarara University of Science and Technology, Ishaka, Uganda, must.ac.ug

**Keywords:** associated factors, early adverse perinatal outcomes, incidence, multicenter prospective cohort, placental abruption, Uganda

## Abstract

**Background and Aims:**

Placental abruption is one of the leading causes of perinatal morbidity and mortality, particularly in low‐ and middle‐income countries. In 2015, the World Health Organization prioritized improving maternal and newborn care to reduce preventable deaths. This study is aimed at determining the proportion of early adverse perinatal outcomes and identifying the factors associated with these outcomes among women with placental abruption delivering at three tertiary hospitals in Uganda.

**Methods:**

This prospective cohort study was conducted from September 30, 2023, to January 30, 2024, at Jinja, Fort Portal, and Mubende Regional Referral Hospitals. A total of 110 consecutive women with clinically diagnosed placental abruption were enrolled in labor suites. Data were collected via pretested questionnaires and analyzed via STATA Version 14.2. Descriptive statistics were used to determine the incidence and describe adverse perinatal outcomes. Associations between variables were assessed using bivariate and multivariate analyses, with a significance level set at *p* < 0.05 and a 95% confidence interval. The results were presented using pie charts, bar graphs, and tables.

**Results:**

Among the 110 women with confirmed placental abruption, 70 (63.6%) experienced at least one early adverse perinatal outcome. The most frequent adverse outcomes were neonatal intensive care unit admission (37.1%), stillbirth (27.1%), neonatal death within 48 h (14.3%), low Apgar score (11.4%), and low birth weight (10.0%). Mothers with abnormal fetal heart rates at admission, either abnormally high (adjusted risk ratio [aRR] = 2.80, 95% CI: 1.61–3.22, *p* = 0.005) or low (aRR = 2.56, 95% CI: 1.50–3.10, *p* = 0.004), were significantly more likely to experience early adverse perinatal outcomes. Mothers who delivered via spontaneous vaginal delivery were less likely to have adverse outcomes (aRR = 0.335, 95% CI: 0.105–0.923, *p* = 0.032). Additionally, mothers with > 50% placental detachment were 8 times more likely to experience adverse outcomes (aRR = 2.59, 95% CI: 1.48–3.13, *p* = 0.005).

**Conclusions:**

Women with placental abruption experienced a high burden of early adverse perinatal outcomes. Abnormal fetal heart rate at admission, mode of delivery, and extent of placental detachment were independently associated with these outcomes. These findings highlight the importance of timely diagnosis, close fetal monitoring, and prompt obstetric management among women presenting with placental abruption.

## 1. Introduction

Placental abruption (PA), defined as the premature separation of a normally implanted placenta before delivery, is one of the most serious obstetric emergencies encountered in clinical practice. Although it complicates only 0.5%–1.0% of pregnancies worldwide, PA remains a major contributor to maternal and perinatal morbidity and mortality because of its sudden onset and potential to rapidly compromise both maternal and fetal wellbeing [1, 2]. The condition is responsible for approximately one‐quarter of cases of antepartum hemorrhage and continues to pose substantial challenges, particularly in low‐resource settings where access to timely emergency obstetric and neonatal care may be limited [3].

The adverse effects of PA arise from disruption of placental function, leading to reduced fetal oxygenation and nutrient transfer. Consequently, pregnancies complicated by PA are at increased risk of preterm birth, low birth weight, birth asphyxia, stillbirth, neonatal death, and long‐term neurodevelopmental impairment among surviving infants [4, 5]. Previous evidence suggests that PA contributes substantially to adverse perinatal outcomes and may account for approximately 10% of preterm births and 10%–20% of perinatal deaths in developed countries [6] . However, adverse outcomes are not solely determined by the occurrence of placental separation. Their severity is influenced by several interconnected factors, including gestational age at delivery, the extent of placental detachment, fetal condition at presentation, and the speed with which appropriate obstetric interventions are initiated [7]. In addition, maternal anemia, fetal distress, low birth weight, and delays in accessing healthcare have consistently been associated with poorer neonatal outcomes in pregnancies complicated by PA [8, 9]. Despite the high burden of PA in sub‐Saharan Africa, context‐specific evidence remains scarce. Consequently, the determinants and outcomes of PA in many African settings are not well understood.

In Uganda, neonatal mortality remains a significant public health concern, with an estimated 27 neonatal deaths per 1000 live births despite ongoing efforts to improve maternal and newborn health services [10]. Persistent barriers, including delayed referrals, shortages of essential medicines and equipment, inadequate neonatal care capacity, and gaps in emergency obstetric services, continue to hinder progress toward improved neonatal survival [11, 12].

Although PA is recognized as an important contributor to adverse perinatal outcomes, there is a paucity of prospective data describing the magnitude of these outcomes and the factors associated with them in Ugandan healthcare settings. A better understanding of the burden and determinants of adverse perinatal outcomes among women with PA is essential for identifying high‐risk pregnancies, improving timely obstetric management, and informing interventions aimed at reducing preventable neonatal morbidity and mortality. Therefore, this study is aimed at determining the proportion of early adverse perinatal outcomes and identifying the associated factors among women with PA delivering at three tertiary hospitals in Uganda.

## 2. Materials and methods

### 2.1. Study Design and Setting

This prospective cohort study was conducted at Jinja, Fort Portal, and Mubende Regional Referral Hospitals (JRRH, FRRH, and MRRH) between September 30, 2023, and January 30, 2024. These tertiary public hospitals are located in Eastern, Western, and Central Uganda, respectively, and serve as the main referral centers for their surrounding districts.

All three hospitals provide comprehensive emergency obstetric and neonatal care services, including cesarean delivery, blood transfusion, newborn resuscitation, and neonatal intensive care. The hospitals are staffed by specialist obstetricians, pediatricians, medical officers, and midwives and receive referrals from lower‐level health facilities across their respective catchment areas. Together, the three sites serve a diverse population and provide an appropriate setting for evaluating perinatal outcomes among women with PA in Uganda.

### 2.2. Study Population

The study included women aged 15–49 years with singleton pregnancies who presented in labor with clinically suspected PA at the participating hospitals. The age range was selected because it corresponds to the conventional definition of women of reproductive age. Pregnant participants younger than 18 years were considered emancipated minors and were eligible to provide independent written informed consent in accordance with the protocol approved by the Research Ethics Committee.

Women with multiple gestations, pre‐existing chronic hypertension defined as hypertension diagnosed before pregnancy or before 20 weeks of gestation diabetes mellitus, renal disease, or pregnancies conceived using assisted reproductive technologies were excluded because these conditions could independently affect perinatal outcomes. Women who developed hypertensive disorders after 20 weeks of gestation, including preeclampsia, remained eligible because these conditions may coexist with PA and were evaluated as potential associated factors.

### 2.3. Sample Size Calculation

The following sample size formula from Daniels WW (1999) was employed:

N=Z2p1−pe2,

where *N* is the required sample size estimate, and *Z* is the critical value for a normal distribution at the 95% confidence level, corresponding to 1.96; *p* is the incidence of early adverse perinatal outcomes, for which a 50% estimated incidence was used due limited data in this field of study. *q* = 1 − *p*. Therefore, *N* = 1.96^2^∗0.5(1 − 0.5)/0.05^2^ = 384.

For a finite population, we adjusted the sample size using the finite population correction factor (Lavrakas, 2008);

The finite population estimate (*N* = 135) was obtained from delivery suite records at Jinja, Fort Portal, and Mubende Regional Referral Hospitals, which documented approximately 135 cases of clinically diagnosed PA during a comparable 4‐month period preceding the study.

nn01+n0−1/N,

where *n*_0_ is the initial sample size (384); *N* is the adjusted sample size (135) at three study sites (JRRH, FRRH and MRRH).

n=384100.07/1135+3841−/=,

where *n* = 100 participants.

Adding 10% loss of follow‐up, the sample size considered in this study became 110.

### 2.4. Study Procedure

Women aged 15–49 years with singleton pregnancies and clinically suspected PA were screened for eligibility upon admission to the labor ward. Trained research assistants explained the study objectives, procedures, potential risks, and benefits in a language understood by each participant. Participants aged 18 years or older provided written informed consent. Pregnant participants younger than 18 years were considered emancipated minors and provided independent written informed consent in accordance with the REC‐approved protocol. Parental or guardian permission and a separate assent procedure were therefore not required. Participant privacy and confidentiality were maintained throughout the study.

PA was initially diagnosed clinically by an attending obstetrician or senior obstetric resident. Women presenting with vaginal bleeding, abdominal pain, uterine tenderness, uterine hypertonicity, nonreassuring fetal status, or unexplained intrauterine fetal demise were considered to have suspected PA. Ultrasonography, when available, supported the clinical assessment but was not required because of its limited sensitivity for PA.

All enrolled women received routine obstetric care according to hospital protocols. After delivery, an experienced obstetrician examined the placenta for retroplacental clots and placental separation. The proportion of the maternal placental surface affected by separation was estimated visually and categorized as < 30%, 30%–50%, or > 50% using a standardized approach across the study hospitals. The same assessment was performed after vaginal and cesarean deliveries.

Only women with PA confirmed by postpartum placental examination were included in the final analysis. Among 125 women with clinically suspected PA, 110 had postpartum confirmation and were analyzed. All newborns received routine neonatal care and were followed for 48 h after birth to assess early adverse perinatal outcomes.

### 2.5. Data Collection Procedure

All eligible participants were given a structured, pretested, interviewer‐administered questionnaire (File S1) to collect information on sociodemographic and obstetric factors related to adverse perinatal outcomes. About 10% of the sample size was selected to pretest the data collection tool at Kampala International University Teaching Hospital to ensure its validity and consistency. The reliability of the tool was confirmed with a Cronbach′s alpha coefficient of 88%, and its validity was ensured through precise and consistent instrumentation

### 2.6. Study Variables

The primary outcome was early adverse perinatal outcome, assessed as a binary variable among women with confirmed PA. Independent variables included sociodemographic characteristics (maternal age, marital status, education level, occupation, residence, and smoking status) and obstetric factors (gravidity, gestational age, antenatal care attendance, mode of delivery, admission fetal heart rate, admission blood pressure, duration of symptoms, and retroplacental clot size).

Early adverse perinatal outcome was defined as the occurrence of at least one of the following: stillbirth, neonatal death within 48 h of birth, neonatal intensive care unit (NICU) admission, low Apgar score (< 7 at 5 min), or low birth weight (< 2500 g). For the composite outcome analysis, participants experiencing one or more of these events were classified as having an adverse perinatal outcome. Individual outcome components were analyzed separately in the descriptive analysis.

**Figure 1:**
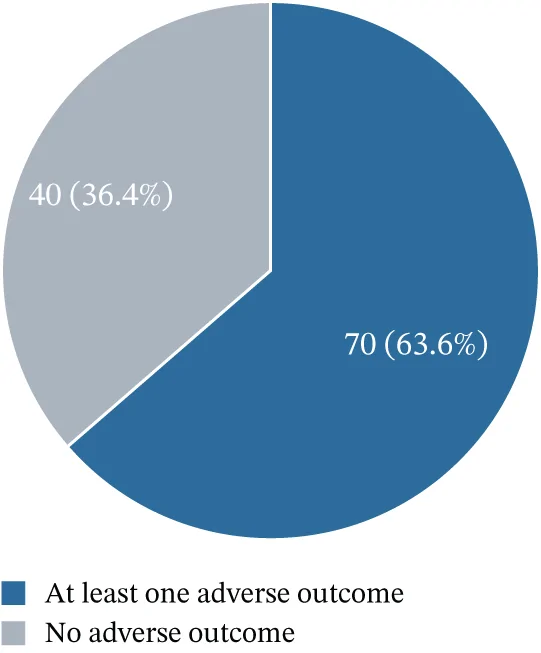
Incidence of early adverse perinatal outcomes among women with confirmed placental abruption in three tertiary hospitals in Uganda.

For descriptive presentation of early adverse perinatal outcomes, neonates who experienced more than one adverse outcome were classified according to the most clinically severe outcome using a hierarchical severity approach, ranked as stillbirth, neonatal death within 48 h, NICU admission, low Apgar score, and low birth weight. Consequently, each neonate contributed only one outcome to the descriptive analysis presented in Figure 1.

### 2.7. Data Quality Control

To ensure data quality, the study employed standardized participant recruitment and data collection procedures. The questionnaire was pretested prior to data collection, and research assistants received training on study objectives, ethical conduct, and data collection methods. The principal investigator and senior research assistants provided continuous supervision and performed daily checks of completed questionnaires for completeness, consistency, and protocol adherence.

### 2.8. Data Management and Analysis

Completed questionnaires were reviewed for completeness and consistency before data entry. Data were entered into Microsoft Excel 2016 for cleaning and coding and subsequently exported to Stata Version 14.2 (StataCorp, College Station, Texas, United States) for analysis.

Participant characteristics were summarized using descriptive statistics. The incidence of early adverse perinatal outcomes was calculated as the proportion of women with confirmed PA who experienced at least one adverse outcome during the follow‐up period. The frequency and distribution of individual adverse perinatal outcomes were also described.

Variables with *p* < 0.20 at bivariable analysis together with variables considered clinically relevant based on previous literature were entered simultaneously into the multivariable log‐binomial regression model using a forced‐entry approach. These variables included maternal education level, occupation, gestational age, antenatal care attendance, predelivery blood pressure, admission fetal heart rate, duration of symptoms before presentation, mode of delivery, and extent of placental detachment. Adjusted risk ratios reported in the final model represent estimates after simultaneous adjustment for these variables.

No formal adjustment for multiple comparisons was performed because the analyses were based on prespecified hypotheses and the primary objective was estimation of adjusted associations rather than hypothesis screening. Results were interpreted considering the magnitude and precision of adjusted risk estimates rather than statistical significance alone.

### 2.9. Ethical Considerations

Ethical approval was obtained from the Bishop Stuart University Research Ethics Committee (BSU‐REC‐2023‐114) before commencement of participant recruitment and data collection. Administrative clearance was obtained from Jinja Regional Referral Hospital, Fort Portal Regional Referral Hospital, and Mubende Regional Referral Hospital. The study was registered with the Uganda National Council for Science and Technology. Written informed consent was obtained from all participants before enrolment after detailed explanation of the study objectives, procedures, risks, and benefits. Participation was voluntary, and participants were free to withdraw at any stage without affecting their medical care. Confidentiality was maintained through the use of coded identifiers and secure storage of study records. The study was conducted according to the principles of the Declaration of Helsinki.

## 3. Results

### 3.1. Sociodemographic and Obstetric Characteristics of the Study Participants

Of the 125 women initially suspected to have PA during the study period, 110 cases were confirmed by placental examination and included in the final analysis. Among the 110 participants, the majority were aged between 20 and 29 years 51 (46.4%), from urban areas 71 (64.5%), were married 75 (68.2%), had secondary education 37 (33.6%), were housewives 46 (41.8%), and identified as Christians 91 (82.7%) (Table 1).

**Table 1:** Sociodemographic and obstetric characteristics of the study participants (*N* = 110).

Variables	Categories	Frequency (*n*)	Percentage (%)
Age	≥ 40	7	6.4
	30–39	46	41.8
	**20–29**	**51**	**46.4**
	< 20	6	5.5

Residence	Rural	39	35.5
	**Urban**	**71**	**64.5**

Marital status	Single	22	20
	Divorced	13	11.8
	**Married**	**75**	**68.2**

Level of education	None	18	16.4
	Primary	35	31.8
	**Secondary**	**37**	**33.6**
	Tertiary	20	18.2

Occupation	**Housewife**	**46**	**41.8**
	Farmer	18	16.4
	Business	29	26.4
	Professional	17	15.5

Religion	Muslim	19	17.3
	**Christian**	**91**	**82.7**

Gravidity	**Multigravida**	**94**	**85.5**
	Primigravida	16	14.5

Gestational age in weeks	**< 37w**	**57**	**51.8**
	≥ 37w	53	48.2

Number of antenatal care visit	None	11	10
	1–2	22	20
	**3–4**	**51**	**46.4**
	> 4	26	23.6

Predelivery blood pressure	Abnormally high	11	10
	Low	29	26.4
	**Normal range**	**70**	**63.6**

Admission fetal heart rate	Abnormally high	23	20.9
	Low	29	26.4
	**Normal range**	**58**	**52.7**

Duration of symptoms	> 8 h	19	17.3
	6–8 h	27	24.5
	**3–5** h	**48**	**43.6**
	1–2 h	2	1.8
	< 1 h	14	12.7

Mode of delivery	SVD	30	27.3
	**Cesarean section**	**80**	**72.7**

Retroplacental clot	> 50%	35	31.8
	30%–50%	30	27.3
	**< 30%**	**45**	**40.9**

*Note:* Boldface denotes the majority of participants.

### 3.2. Incidence of early adverse perinatal outcomes among women with confirmed PA in three tertiary hospitals in Uganda (*N* = 110).

Among the 110 women with confirmed PA, 70 (63.6%) experienced at least one early adverse perinatal outcome (Figure 1).

Early adverse perinatal outcomes among women with confirmed PA delivering at three tertiary hospitals in Uganda (*N* = 70)

**Figure 2:**
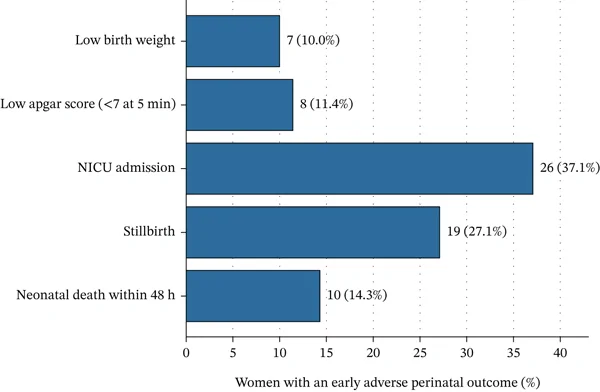
Distribution of the most clinically severe early adverse perinatal outcome among neonates born to women with placental abruption (*n* = 70).

Out of 70 women who had adverse outcomes, NICU admission represented 26 (37.1%), followed by stillbirth in 19 (27.1%), neonatal death within 48 h in 10 (14.3%) and a low Apgar score in 8 (11.4%). The least common outcome was low birth weight which was observed in 7 (10%) of all cases (Figure 2).

For descriptive purposes, each neonate experiencing one or more adverse outcomes was classified according to the most clinically severe outcome. Consequently, the categories presented in Figure 2 are mutually exclusive and sum to the total number of neonates with at least one early adverse perinatal outcome.

### 3.3. Bivariable and multivariable analyses of factors associated with early adverse perinatal outcomes among women with confirmed PA delivering at three tertiary hospitals in Uganda (*N* = 110).

Table 2 presents the bivariable and multivariable log‐binomial regression analyses of factors associated with early adverse perinatal outcomes among women with confirmed PA. Admission fetal heart rate, mode of delivery, and the extent of placental detachment remained independently associated with early adverse perinatal outcomes after adjustment for potential confounders.

**Table 2:** Bivariable and multivariable analyses of factors associated with early adverse perinatal outcomes among women with confirmed placental abruption delivering at three tertiary hospitals in Uganda (*N* = 110).

Variables	Categories	cRR (95% CI)	*p*	aRR (95% CI)	*p*
Level of education	None	1.54 (0.62–2.54)	0.301	0.46 (0.061–1.90)	0.36
	Primary	1.72 (0.62–2.53)	0.116	1.05 (0.25–2.39)	0.942
	Secondary	1.38 (0.63–2.26)	0.376	0.95 (0.23–2.26)	0.933
	Tertiary	Ref			

Occupation	Housewife	1.94 (1.02–2.72)	0.044	1.93 (0.29–3.17)	0.392
	Farmer	1.83 (0.79–2.77)	0.135	1.46 (0.12–3.14)	0.698
	Business	1.79 (0.84–2.66)	0.112	2.19 (0.39–3.22)	0.274
	Professional	Ref			

Gestational age	< 37 weeks	2.00 (1.33–2.58)	0.003	1.72 (0.93–2.48)	0.078
	≥ 37 weeks	Ref			

Number of ANC visit	None	2.20 (0.86–3.05)	0.085	0.63 (0.04–2.76)	0.691
	1–2	1.98 (0.98–2.79)	0.057	0.82 (0.14–2.35)	0.789
	3–4	1.33 (0.68–2.11)	0.367	0.87 (0.23–2.06)	0.795
	> 4	Ref			

Predelivery BP	Abnormally high	2.20 (0.93–3.02)	0.066	2.17 (0.62–3.13)	0.171
	Low	2.63 (1.69–3.3)	0.001	1.56 (0.45–2.77)	0.416
	Normal range	Ref			

Admission FHR	Abnormally high	2.88 (1.92–3.22)	0.001	2.80 (1.61–3.22)	**0.005**
	Low	2.64 (1.80–3.08)	<0.001	2.56 (1.50–3.10)	**0.004**
	Normal range	Ref			

Duration of symptoms	> 8 h	2.68 (1.47–3.18)	0.007	2.37 (0.765–3.18)	0.104
	6–8 h	1.02 (0.957–1.79)	0.064	1.45 (0.482–2.73)	0.517
	3–5 h	1.88 (0.905–2.72)	0.082	0.94 (0.241–2.21)	0.911
	1–2 h	1.45 (0.125–3.13)	0.699	0.77 (0.014–3.32)	0.923
	< 1 h	Ref			

Mode of delivery	SVD	0.54 (0.255–1.05)	0.072	0.33 (0.105–0.923)	**0.032**
	Cesarean section	Ref			
Retroplacental clot	> 50%	2.87 (2.08–3.19)	<0.001	2.59 (1.48–3.13)	**0.005**
	30–50%	1.68 (0.94–2.41)	0.076	1.29 (0.52–2.29)	0.538
	< 30%	Ref			

*Note:* bold values indicate *p* < 0.05.

Abbreviations: aRR, adjusted risk ratio; CI, confidence interval; cRR, crude risk ratio.

Compared with women whose admission fetal heart rate was within the normal range, those presenting with fetal tachycardia (aRR = 2.80, 95% CI: 1.61–3.22, *p* = 0.005) or fetal bradycardia (aRR = 2.56, 95% CI: 1.50–3.10, *p* = 0.004) had a significantly higher risk of early adverse perinatal outcomes. Conversely, spontaneous vaginal delivery was associated with a 66.5% lower risk of adverse outcomes compared with cesarean delivery (aRR = 0.34, 95% CI: 0.11–0.92, *p* = 0.032). Women with placental detachment involving more than 50% of the placental surface had a significantly increased risk of early adverse perinatal outcomes compared with those with less than 30% detachment (aRR = 2.59, 95% CI: 1.48–3.13, *p* = 0.005).

## 4. Discussion

In this multicenter prospective cohort study, 70 of 110 women with confirmed PA experienced at least one early adverse perinatal outcome, corresponding to an incidence of 63.6%. This incidence is comparable to the 56.4% reported in Ethiopia and the 59.3% reported in Pakistan [8, 13]. However, it exceeds the 30% reported at Jimma University Medical Center and the approximately 15% perinatal mortality estimate reported globally [5, 14]. The higher burden observed in our cohort may reflect more severe disease at presentation, as 59.1% of participants had placental detachment exceeding 30% of the placental surface, in addition to delays in accessing definitive obstetric care. Extensive placental separation compromises fetal oxygenation and nutrient transfer, thereby increasing the risk of fetal compromise and adverse neonatal outcomes. These findings underscore the importance of timely diagnosis, referral, and intervention in pregnancies complicated by PA.

Among women who experienced adverse outcomes, NICU admission was the most common event, accounting for 37.1% of cases, followed by stillbirth (27.1%), neonatal death within 48 h (14.3%), low Apgar score at 5 min (11.4%), and low birth weight (10.0%). The combined proportion of stillbirths and early neonatal deaths was 41.4%, which was lower than the 56.4% reported in Ethiopia but substantially higher than the 8.7% perinatal mortality rate reported in Dubai [8, 15] These differences may be explained by variations in the extent of placental separation, gestational age at delivery, timeliness of intervention, and the availability of neonatal intensive care services. Nevertheless, the findings highlight the substantial contribution of PA to preventable perinatal mortality in resource‐constrained settings.

Abnormal fetal heart rate at admission was independently associated with adverse perinatal outcomes. Compared with women whose fetal heart rate was within the normal range, those presenting with fetal tachycardia and bradycardia had a 2.8‐fold (aRR = 2.80, 95% CI: 1.61–3.22) and 2.6‐fold (aRR = 2.56, 95% CI: 1.50–3.10) higher risk of adverse outcomes, respectively. This finding is biologically plausible because PA impairs uteroplacental perfusion, leading to fetal hypoxia that manifests as abnormal fetal heart rate patterns. Similar associations have been reported in previous studies, where fetal heart rate abnormalities were strong predictors of fetal compromise and poor neonatal outcomes [14, 16]

Women who delivered vaginally had a lower risk of adverse perinatal outcomes than those delivered by cesarean section (aRR = 0.34, 95% CI: 0.11–0.92). However, this association should be interpreted with caution, as mode of delivery may act as a marker of underlying disease severity rather than a causal determinant of outcome. Women requiring emergency cesarean delivery are more likely to present with severe PA, fetal compromise, or other obstetric emergencies that independently increase the risk of adverse perinatal outcomes. Therefore, the possibility of residual confounding and indication bias cannot be excluded.

The extent of placental detachment was another important predictor of outcome. Women with placental separation involving more than 50% of the placental surface had a 2.6‐fold higher risk of adverse perinatal outcomes than those with less than 30% separation (aRR = 2.59, 95% CI: 1.48–3.13). This finding is consistent with previous studies demonstrating that larger abruptions are associated with increased neonatal morbidity, prolonged NICU admission, and higher perinatal mortality [2, 9]. Because fetal survival is closely linked to both the extent of placental separation and gestational age at delivery, prompt recognition and timely intervention remain critical to improving neonatal outcomes.

Overall, these findings emphasize the need to strengthen fetal surveillance, improve referral systems, and ensure timely access to emergency obstetric and neonatal care. Such interventions are likely to reduce the substantial burden of adverse perinatal outcomes associated with PA in Uganda and similar low‐resource settings.

Recent evidence has also highlighted the potential role of inexpensive laboratory biomarkers in identifying pregnancies at increased risk of PA and adverse perinatal outcomes. Reported that a lower first‐trimester hemoglobin, albumin, lymphocyte, and platelet (HALP) score was significantly associated with PA and composite adverse perinatal outcomes OzdalBurcu et al. [17]. Given that HALP is derived from routinely available laboratory parameters, it may represent a practical risk stratification tool in low‐resource settings and warrants further evaluation in African populations.

During study planning, no published Ugandan prospective data were available on the incidence of early adverse perinatal outcomes among women with PA. Therefore, a conservative prevalence estimate of 50% was used for sample size calculation following standard epidemiological recommendations. Using *p* = 0.50 maximizes the required sample size and ensures adequate statistical power when the true outcome frequency is unknown. Although the observed incidence in the present study was 63.6%, this does not invalidate the original sample size calculation because the prevalence estimate was selected a priori before participant recruitment.

### 4.1. Strengths and Limitations

This study provides important evidence on the burden and determinants of adverse perinatal outcomes among women with PA in Uganda. To the best of our knowledge, it is the first prospective multicenter study in the country to examine early adverse perinatal outcomes in this high‐risk population. By recruiting participants from three tertiary referral hospitals serving diverse populations across different regions of Uganda, the study enhances the generalizability of its findings and provides context‐specific evidence to inform clinical practice and future research.

Several limitations should be considered when interpreting the results. Neonates were followed for 48 h after birth, which allowed the study to capture immediate complications associated with PA. However, adverse outcomes occurring later during the early neonatal period may have been missed, potentially resulting in an underestimation of the true burden of neonatal morbidity and mortality. In addition, the extent of placental detachment was assessed visually after delivery. Although a standardized assessment approach was used across all study sites, some degree of observer variability cannot be entirely excluded. Finally, the absence of a comparison group of women without PA limited our ability to directly quantify the excess risk attributable to PA.

Despite these limitations, the prospective design, multicenter setting, standardized data collection procedures, and systematic follow‐up of neonates strengthen the reliability of the findings. The study offers valuable insights into the magnitude of adverse perinatal outcomes associated with PA and identifies clinically important factors that may assist in the early recognition of pregnancies at increased risk of poor neonatal outcomes.

Neonates were followed for the first 48 h after birth because this represented the period during which all newborns remained hospitalized and could be assessed consistently across the three study hospitals. Although this approach enabled complete ascertainment of immediate neonatal outcomes, adverse events occurring after discharge but within the conventional 7‐day early neonatal period may not have been captured. Consequently, the overall burden of early neonatal morbidity and mortality may have been underestimated.

## 5. Conclusions and Recommendations

PA was associated with a high burden of early adverse perinatal outcomes among women delivering at three tertiary hospitals in Uganda. NICU admission, stillbirth, and early neonatal death were the most frequent adverse outcomes observed. Abnormal fetal heart rate at admission and extensive placental detachment were independently associated with an increased risk of adverse perinatal outcomes, highlighting the importance of early recognition of fetal compromise and disease severity. These findings emphasize the need to strengthen antenatal risk assessment, timely referral, intrapartum fetal monitoring, and access to emergency obstetric and neonatal care to improve perinatal outcomes among pregnancies complicated by PA.

**Nomenclature**
PA: placental abruption
JRRH: Jinja Regional Referral Hospital
FRRH: Fort Portal Regional Referral Hospital
MRRH: Mubende Regional Referral Hospital
NICU: neonatal intensive care unit
WHO: World Health Organization
REC: Research Ethics Committee
LMICs: low‐ and middle‐income countries

## Author Contributions

I.O.S. conceptualized the proposal and was actively involved in data collection and analysis. T.H. and M.P.S.I. played key roles in data collection, entry, analysis, and manuscript drafting. T.N. and R.K. contributed to refining the proposal, supporting the analysis process, and revising the manuscript.

## Funding

No funding was received for this manuscript.

## Disclosure

All authors have read and approved the final version of the manuscript. Theoneste Hakizimana had full access to all study data and takes complete responsibility for the integrity of the data and the accuracy of the data analysis. Theoneste Hakizimana, the manuscript guarantor, affirms that this manuscript is an honest, accurate, and transparent account of the study being reported; that no important aspects of the study have been omitted; and that any discrepancies from the study as originally planned have been explained.

## Ethics Statement

Ethical approval was obtained from the Bishop Stuart University Research Ethics Committee (BSU‐REC‐2023‐114), and the study was registered with the Uganda National Council for Science and Technology. Administrative permission was obtained from Jinja, Fort Portal, and Mubende Regional Referral Hospitals before participant recruitment. All participants provided written informed consent before enrolment. Participants aged 18 years or older consented independently. Pregnant participants younger than 18 years were classified as emancipated minors and provided independent written informed consent in accordance with the REC‐approved protocol; parental or guardian permission and a separate assent procedure were therefore not required. Participation was voluntary, and confidentiality was maintained using coded study identifiers and secure data storage. The study was conducted in accordance with the Declaration of Helsinki and applicable Ugandan ethical guidelines.

## Consent

The authors have nothing to report.

## Conflicts of Interest

The authors declare no conflicts of interest.

## Supporting information


**Supporting Information** Additional supporting information can be found online in the Supporting Information section. File S1: Data collection questionnaire used to obtain sociodemographic, obstetric, and early perinatal outcome information from study participants.

## Data Availability

The datasets generated and analyzed during the current study are available from the corresponding author upon reasonable request. Data are not publicly available because they contain information that could compromise participant confidentiality (theonestehakizimana5@gmail.com).
