# Supplementary material for: Early Adverse Perinatal Outcomes Among Women With Placental Abruption: A Multicenter Prospective Cohort Study in Uganda
Source: J Pregnancy. 2026 Sep 21;2026:6074764. doi: 10.1155/jp/6074764 (PMC13591998; doi:10.1155/jp/6074764)
Supplement: Supplementary file 1 [file JP-2026-6074764-s001.docx]

**S1 Appendix. Data collection questionnaire**

***Incidence and Factors Associated with Early Adverse Perinatal Outcomes among Women with Placental Abruption Delivering at Three Tertiary Hospitals in Uganda: A Multicenter Prospective Cohort Study***

Patient serial number…………………….	Date………………………………

Telephone contact………………………

Next of kin………………………………	Phone contact…………………………….

Date of admission: ……………………………………………

Date of delivery: ………………………………………………

1. Place of residence
   A) Rural
   B) Urban
2. How old are you?

<20 …………………….

20-29……………………

30 -39…………………...

≥40………………………

3. Level of education
A) None
B) Primary
C) Secondary
D) Institution/college
E) Tertiary
4. Religion
A) Christian
B) Muslim
5. Marital Status
A) Married
B) Single
C) Cohabiting
D) Widowed
E) Divorced

6. What is your occupation?

A) Housewife

B) Farmer

C) Professional

D) Business

7. Did you attend ANC in this recent pregnancy?

A) Yes

B) No

8. Parity…………………...

9. Gravidity………………...

10. Gestational age in weeks………….

11. Maternal pre delivery BP

A)	Low

B)	Normal range

C) abnormally high

12. Admission FHR

A) Low

B) Normal range

C) abnormally High

13. ANC attendance

A) No

B) Yes

14. how many times

A) Not attended

B) 1-2

C) 3-4

D) >4

15. Mode of delivery

A) SVD

B) Cesarean section

16.maternal effects

a) Cesarean section

c) blood transfusion

d) Admission in ICU

e) Hysterectomy

f) Primary PPH

g) Hypovolemic shock

h) Maternal death

i) None of above

17. Presenting signs and symptoms?

A) abdominal pain

B) PV bleeding

C) abdominal pain and PV bleeding

D) Hypertonic Uterus

E) Fetal distress

F) Others

18. Duration (onset of symptoms) above

A) <2hours

B) 2-4hours

C) 4-6hours

D) 6-12hours

E) >12hours

**Perinatal outcomes 1^st^ min**

19. APGAR score

A) <7
B) ≥7

20. APGAR score 5^th^ min

A) < 7

B) ≥7

21.Lowbirthweight

A) Yes

B) No

22. Small for GA

A) Yes

B) No

23. Stillbirth

A) Yes

B) No

24) NICU admission

A) Yes

B) No

25) Neonatal death in 48hours

A) Yes

B) No

26) Retroplacental clot

A) <30%
B) 30-50%
C) ≥ 50%
